# In Situ Construction of Thermotropic Shape Memory Polymer in Wood for Enhancing Its Dimensional Stability

**DOI:** 10.3390/polym14040738

**Published:** 2022-02-14

**Authors:** Wenhao Zhang, Jianchao Zhou, Zhijin Cao, Xinxing Wu, Hui Wang, Shuaibo Han, Yan Zhang, Fangli Sun, Ting Zhang

**Affiliations:** 1 College of Chemistry and Materials Engineering, National Engineering & Technology Research Center for the Comprehensive Utilization of Wood-Based Resources, Zhejiang A&F University, Hangzhou 311300, China; 2019104041013@stu.zafu.edu.cn (W.Z.); 2020104041019@stu.zafu.edu.cn (J.Z.); 2020604021002@stu.zafu.edu.cn (Z.C.); xinxingwu@zafu.edu.cn (X.W.); wanghui@zafu.edu.cn (H.W.); shuaibohan@zafu.edu.cn (S.H.); 2 Xilinmen Furniture Co., Ltd., Shaoxing 312000, China; t.zhang@sleemon.cn

**Keywords:** shape memory polymer, wood, in situ construction, dimension stability, anti-cracking

## Abstract

The extension of wood to a wider field has been restrained significantly due to its dimensional instability that arises from variation in moisture content, which in turn brings about the risk of cracking, warping or distortion. This work proposed a novel strategy to stabilize wood by means of the in situ construction of a thermotropic shape memory polymer (SMP) inside wood. The cross-linked copolymer network (PMP) with good shape memory behavior was first investigated based on the reaction of methyl methacrylate (MMA) and polyethylene glycol diacrylate (PEGDA) in a water/ethanol solution; then, the PMP was constructed inside wood via vacuum-pressure impregnation and in situ polymerization. The weight gain, volume increment and morphology observations clearly revealed that the PMP was mainly present in wood cell lumens, cell walls and pits. The presence of PMP significantly enhanced the dimensional stability of and reduced the cracks in wood. The desirable shape recovery abilities of PMP under heating-cooling cycles were considered to be the main reasons for wood dimensional stabilization, because it could counteract the internal stress or retard the shrinkage of cell walls once water was evaporated from the wood. This study provided a novel and reliable approach for wood modification.

## 1. Introduction

Wood is one of the most abundant renewable materials and is widely used in construction, decoration and furniture applications [1,2,3]. However, as a hygroscopic material, wood is sensitive to water, fungi, insects, etc., which restrains its wider applications [4,5]. The variation in moisture content causes wood to swell and shrink in various degrees, resulting in deformation and cracking [6,7]. Among the effects of these various defects, cracking is the most notable, as it is related to the mechanical performance and decay resistance of wood, raising safety concerns. To prolong the service life of wood, enhancing its dimensional stability by means of modification holds many potential prospects [8,9].

Different methods, including thermal, physical and chemical treatment, have been employed to improve the dimensional stability of wood. Among them, chemical treatment has been proven to be an efficient way. Various chemical reagents have been utilized, such as vinyl monomers, formaldehyde-based reagents, epoxy resins, furfuryl alcohol, ε-caprolactone, polyester, polyethylene glycol (PEG), etc. [10,11,12,13,14]. Most of these chemicals are designed to block the cell cavity or swell the cell wall without environmental responses. However, due to the flexible changes in environmental factors, it would be more interesting and meaningful to impregnate wood with intelligent responsive materials [15,16].

Shape memory polymer (SMP), a type of smart material, can sense and respond to environmental changes (such as temperature, light, electricity, pH, etc.) by adjusting its state to a predetermined shape [17,18]. The thermally-induced SMP possesses the advantages of easy processibility, good shape memory performance and tunable thermal and mechanical properties, and is the most extensively studied type of SMP [19]. It is generally composed of a stationary and reversible phase and can soften and harden reversibly at a specific temperature. The function of the stationary phase is to memorize and restore the initial form, while the reversible phase completes the deformation and fixation [20,21]. When the environmental temperature exceeds its transition temperature (*T*_trans_), i.e., the glass transition (*T*_g_) or melting temperature (*T*_m_), the SMP becomes soft and deforms under external force. The shape is unchangeable after cooling, and the stress of the material is “frozen”. When the deformed polymer is heated above the transition temperature again, the internal stress in the material is released, driving the malformed polymer back to its original shape [22,23,24].

Many kinds of SMPs based on thermoplastic, thermosetting polymers or blends, such as poly(methyl methacrylate) (PMMA), poly(ε-caprolactone) (PCL), polyurethane, epoxy resins, etc., have been reported in literature [25,26,27,28]. They are widely employed in various fields involving aerospace, biomedicine, textile, household supplies, etc. Nevertheless, to the best of our knowledge, the effect of SMP on solid wood stabilization has not been reported yet thus far. For wood used outdoors, it is of great significance to reveal the response mechanism of SMP on the dimensional stability and durability of solid wood under the combined action of moisture content and temperature.

To construct SMP with the desired shape memory behavior inside wood, low molecular weight monomers or polymers should be selected and impregnated into wood to achieve good penetration and even distribution [29,30]. Polyethylene glycol (PEG), with a lower molecular weight, easily enters into cell walls and swells them, thereby improving the dimensional stability of the wood; however, it is leachable under moisture conditions [31,32]. The functional vinyl polyethylene glycol (PEGDA), as reported by Dong et al., can graft to previously acrylated poplar wood (performed via the reaction between methacryloyl chloride and the hydroxyl groups of the cell wall) via copolymerization to improve its durability [33]. In addition, Yakacki et al. reported on shape-memory networks with tailored thermomechanics that were created via the compolymerization of poly(ethylene glycol) dimethacrylate (PEGDMA) and methyl methacrylate (MMA) [34]. Considering the cracks caused by fast moisture loss under high temperature, this study aimed to establish a thermo-induced SMP with a low transition temperature and high shape memory recovery ratio based on the reaction of methyl methacrylate (MMA) and polyethylene glycol diacrylate (PEGDA) in a water/ethanol solution. Such a reactive solution can readily enter into the porous structures of wood via vacuum-pressure impregnation and polymerize to form SMP in wood, enhancing its dimensional stability.

## 2. Materials and Methods

### 2.1. Materials

Methyl methacrylate (MMA) and poly(ethylene glycol) diacrylate (PEGDA, *M*_n_ = 400 g/mol) were purchased from Aladdin Co., Ltd. (Shanghai, China). Ethylene glycol dimethyl acrylate (EGDMA) was provided by Macklin Co., Ltd. (Shanghai, China). Ammonium persulfate (APS) and ethanol were purchased from Sinopharm Chemical Reagent Co., Ltd. (Shanghai, China). Poplar (*Populus tomentosa*), obtained from a fast-growing plantation located in Shanxi Province, China, was processed into standard radial, tangential and longitudinal sections with dimensions of 20 mm × 20 mm × 20 mm for the dimensional stability test. All the wood blocks were dried using the following procedure: 60 °C for 2 h, 80 °C for 2 h and 105 °C for 4 h.

### 2.2. Synthesis and Performance Evaluation of MMA/PEGDA Copolymer (PMP)

The cross-linked MMA/PEGDA copolymer (simplified as PMP) was synthesized using the following procedure. Firstly, a mixture of water and ethanol was prepared as the solvent at a volume ratio of 1:1. Then, the monomers MMA and PEGDA at a weight percent of 28% were dissolved in the prepared solvent. Subsequently, 2% APS and 4% EGDMA, calculated as the total mass ratio of monomers, were added as the initiator and crosslinker, respectively. After stirring vigorously, a homogeneous and transparent mixture was obtained which was then heated to 80 °C and reacted at the same temperature for 1 h. The obtained polymer sample was dried at 60 °C for 24 h in a vacuum oven to completely remove the solvents. The residual monomers were removed via extraction with ethanol. For comparison, the homopolymers of PMMA and P(PEGDA) were also prepared under the same conditions. To confirm the cross-linking reaction in the final PMP, tetrahydrofuran (THF) and 10 wt.% potassium hydroxide ethanol solution (simplified as KOH solution) were selected to remove the homopolymers (P(PEGDA) and PMMA) successively.

The water absorption capacity (*P_w_*) and volume swelling capacity (*P_v_*) of PMP were tested on three samples with dimensions of 10 mm × 10 mm × 2 mm. Three cycles of water immersion and oven-drying were conducted. In every cycle, the samples were soaked in distilled water at room temperature (around 25 °C) for 24 h, followed by oven-drying at 60 °C to a constant weight [35,36]. The volume (*V*, cm^3^) and mass (*M*, g) of the samples were measured in each cycle. *P_w_* and *P_v_* were calculated according to Equations (1) and (2), respectively.

(1) $$P_{w}=\frac{M_{1n}-M_{0n}}{M_{0n}}\times 100\%$$

(2) $$P_{v}=\frac{V_{1n}-V_{0n}}{V_{0n}}\times 100\%$$

In the above equations, *M*_0*n*_ and *V*_0*n*_ represent the mass and volume of the samples after oven-drying, while *M*_1*n*_ and *V*_1*n*_ denote the mass and volume of the samples after 24 h of water immersion in the *n*th cycle, *n* = 1, 2, 3.

The shape memory performance test of PMP was conducted on specimens with dimensions of 50 mm × 5 mm × 2 mm. Firstly, the flat specimens were heated to 60 °C, then processed into an L-shape by hand while maintaining the temperature. An L-shape with an angle of 90 degrees was ensured by a ruler and protractor. Subsequently, the specimen was transferred into ice water (0 °C) for 10 s to obtain a temporarily fixed L-shape. After that, some of the specimens were placed at ambient temperature for 24 h to record the final angle *A*_1_ and calculate the shape fixation rate. Other L-shape specimens were taken to investigate the shape recovery behavior. The evolution of the L-shaped recovery angle *A*_2_ was measured at 60 °C at intervals of 45 s. A total of 225 s of recovery behavior was recorded for a cycle. The shape memory recovery test was conducted in 4 cycles for each sample with 3 replicates for each treatment. The shape fixation rate *R_f_* and shape recovery rate *R_r_* were calculated according to Equations (3) and (4), respectively.

(3) $$R_{f}=\frac{180-A_{1}}{90}\times 100\%$$

(4) $$R_{r}=\frac{A_{2}-90}{90}\times 100\%$$

### 2.3. Construction of PMP in Wood

The reactive solution mentioned above was impregnated into wood blocks under vacuum (0.06 MPa) for 15 min and then impregnated under pressure (0.8 MPa) for 30 min, with six replicates for each treatment. To obtain the good penetration of the reactive chemicals and reveal the dimensional stability of the wood under various test conditions, the wood blocks were sourced from the sapwood zone and processed into standard radial, tangential and longitudinal section blocks.

After weighing, the impregnated wood blocks were heated at 80 °C for 1 h to complete the polymerization of monomers. Subsequently, the blocks were dried via the programmed procedures (60 °C for 2 h, 80 °C for 2 h and 105 °C for 4 h) to an absolute dry state, with the moisture content (MC) being designated as MC_0_. After cooling, the weight and volume were determined based on the volume percent gain (*VPG*, %) and weight percent gain (*WPG*, %), which were calculated according to Equations (5) and (6), respectively. The blocks without any treatment were also subjected to the same drying and cooling steps as controls.

(5) $$VPG=\frac{V_{T}-V_{0}}{V_{0}}\times 100\%$$

(6) $$WPG=\frac{W_{T}-W_{0}}{W_{0}}\times 100\%$$

In the above equations, *V*_0_ and *V_T_* are the volume (cm^3^) of the wood specimens before and after treatment, respectively, and *W*_0_ and *W_T_* are the weight (g) of the specimens before and after treatment, respectively.

### 2.4. Dimensional Stability of Wood

To evaluate the dimensional stability of the wood under changing humidity or a rainy-sunny environment, three moistening-drying (M-D) and soaking-drying (S-D) cycles were conducted. Six treated and untreated replicate blocks were prepared for each cycle test. In the M-D test, the volumes (*V*_0_) and weight (*M*_0_) of the samples dried following the procedure mentioned above were firstly determined, then the samples were conditioned in an incubator set at a relative humidity of 85 ± 5% and temperature of 25 ± 2 °C for 3 days, followed by another measurement of the volume (*V_t_*) and weight (*M_t_*). These drying and moistening procedures were repeated to obtain three cycles of variations in the weight and volume [37]. The S-D cycles for the blocks were similar to the M-D cycles, except for replacing the moistening procedure with water immersion. The percentage of swelling (*S*) upon changed moisture content as well as the anti-swelling efficiency (*ASE*) were calculated according to Equations (7) and (8), respectively.

(7) $$S=\frac{V_{tn}-V_{0n}}{V_{0n}}\times 100\%$$

(8) $$ASE=\frac{S_{0}-S}{S_{0}}\times 100\%$$

In the above equations, *V*_0*n*_ and *V_tn_* are the volume of the test block after the *n*th drying and moistening/soaking, respectively; *S*_0_ and *S* are the swelling of the untreated wood (W) and modified wood (MW), respectively; and *ASE* is the anti-swelling efficiency of the modified wood after the *n*th moistening/soaking.

### 2.5. Effect of the Shape Memory Properties of PMP on Wood Dimensional Stability

Treated and untreated blocks of six replicates, after being dried to MC_0_, were conditioned near fiber saturation point (FSP) moisture content (MC_1_) and the volume at this state was recorded as V_1_. The blocks were then heated to 60 °C and maintained for 2 h to measure the dimensions V_2_ and MC_2_, followed by 1 h-room temperature (25 °C) storage and the determination of V_3_ and MC_3_. Subsequently, the temperature was increased to 60 °C again to record the dimensions V_4_ and MC_4_. The blocks went through a total of five cycles of 1 h at room temperature and heating for 2 h at 60 °C. The shrinkage of the blocks was calculated according to Equation (9).

(9) $$Shrinkage=\frac{V_{n}-V_{n+1}}{V_{n}}\times 100\%$$

In Equation (9), *V_n_* is the volume of the blocks after 1 h at room temperature and *V_n_*_+1_ is the volume after heating for 2 h at 60 °C, *n* = 1, 3, 5.

### 2.6. Characterizations

Fourier transform infrared (FT-IR) spectra of P(PEGDA), PMMA and PMP were constructed using a Shimadzu IR Spectrophotometer (IRPrestige-21, Shimadzu, Japan) in transmission mode and the KBr pellet method in a wavenumber of 400 to 4000 cm^−1^ with a 4 cm^−1^ resolution and 32 scans.

Thermal analyses were performed with differential scanning calorimetry (DSC, TA Instruments Q2000) under a nitrogen atmosphere from −75 °C to 150 °C with a scanning rate of 10 °C/min. Before the test, the sample was heated to 150 °C and then cooled to −50 °C at a cooling rate of 10 °C/min to eliminate thermal history.

The morphological structures of the fractured surfaces of a sample of PMP and the modified wood were investigated using a scanning electron microscope (SEM; TM3030, HITACHI, Tokyo, Japan).

## 3. Results and Discussion

### 3.1. Polymerization of MMA and PEGDA

**Figure 1:**
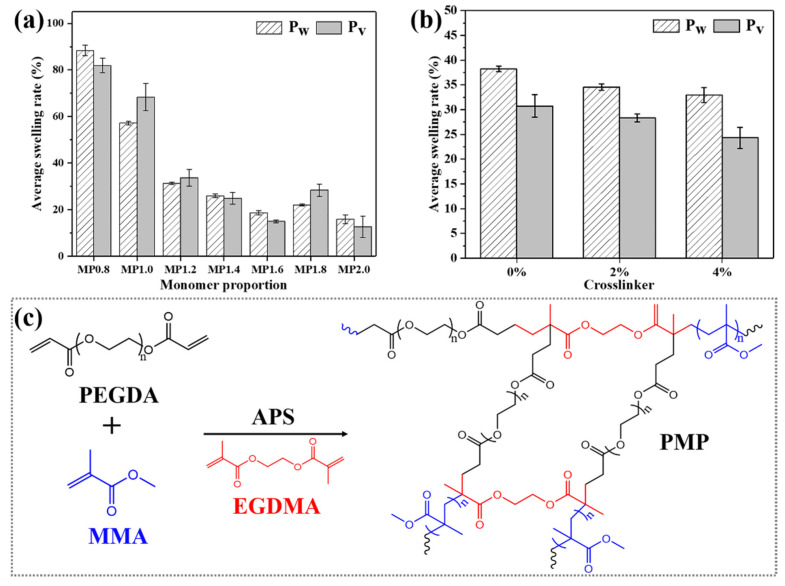
The effect of monomer proportion (**a**) and crosslinker (**b**) on the average swelling rate of PMP. (**c**) Schematic presentation of the MMA/PEGDA cross-linked copolymer network.

**Figure 2:**
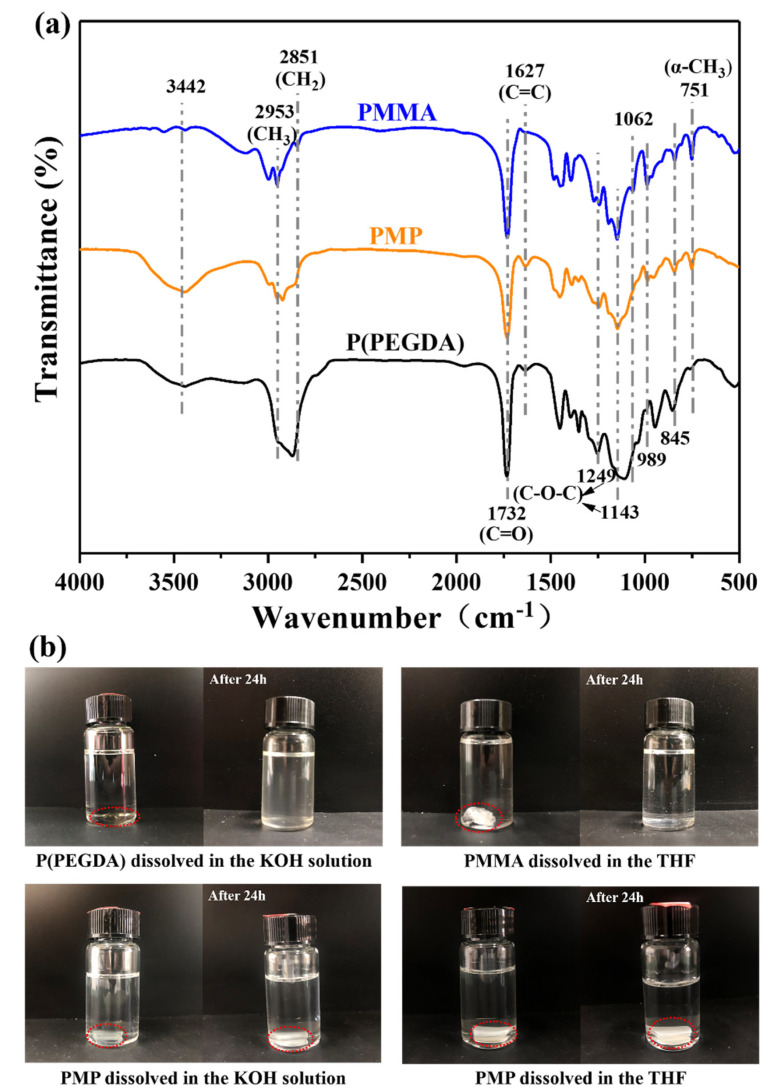
(**a**) FTIR spectra and (**b)** dissolution performance in different solvents for samples of PMMA, P(PEGDA) and PMP. The samples are indicated by red circles.

The cross-linked copolymer PMP with PMMA as the hard segments and P(PEGDA) as the soft segments was prepared. To obtain suitable PMP for wood modification, the mass ratio of MMA to PEGDA was firstly investigated as 0.8:1 (MP0.8), 1.0:1 (MP1.0), 1.2:1 (MP1.2), 1.4:1 (MP1.4), 1.6:1 (MP1.6) and 1.8:1 (MP1.8). The swelling rate of PMP dropped rapidly with the increasing ratio of MMA to PEGDA (see Figure 1a), which could be ascribed to the hydrophobicity of the MMA segment designed to improve the water and moisture resistance. However, when the mass ratio of MMA to PEGDA exceeded 1.6:1, the swelling rate no longer declined. Although the ratio of 1.6:1 was the best option from the aspect of the swelling ratio, serious phase separation occurred on the final PMP product; accordingly, 1.4:1 was applied as the ratio of monomer MMA to PEGDA in the following studies. The obtained PMP was an even, translucent, hard and tough polymer in comparison with the rough, white and brittle PMMA as well as the transparent, soft and weak P(PEGDA). MMA was likely to copolymerize with PEGDA, and the copolymers also tended to cross-link via EGDMA to provide additional restraint in volume swelling and weight increment. The average volume swelling and weight increment declined with the increased amount of crosslinker (Figure 1b). A schematic representation of the MMA/PEGDA cross-linked copolymer network formation is presented in Figure 1c. Such copolymerization and cross-linking reactions were clearly evidenced by the results of the FTIR and dissolution measurements (see Figure 2).

The FTIR spectra for PMMA, P(PEGDA) and PMP are presented in Figure 2a. It is worth noting that the characteristic peaks from 2953 cm^−1^ to 2851 cm^−1^, corresponding to the stretching vibration of −CH_3_ and −CH_2_, changed significantly, which was caused by the copolymerization and cross-linking of MMA and PEGDA. The typical absorptions of 1479 and 751 cm^−1^ assigned to the −CH_2_ bending vibration and −CH_3_ deformation vibration of PMMA appeared in PMP, suggesting the inclusion of MMA in PMP [38,39]. The presence of peaks at 3442 cm^−1^ and 1627 cm^−1^ in PMP were attributed to the stretching and bending vibrations of the −OH groups from the absorption moisture, which is characteristic of the PEG segment, indicating that PEGDA was bonded in PMP [40,41]. The sharp peak at 1732 cm^−1^ was assigned to carbonyl group stretching, and the peaks at 1249 cm^−1^ and 1143 cm^−1^ belonged to C−O−C, which existed in PMMA, P(PEGDA) and PMP. The band at 989 cm^−1^ together with the bands at 1062 cm^−1^ and 845 cm^−1^ were the characteristic absorption vibrations of PMMA [39]. The FTIR results confirmed the presence of PMMA and PEGDA segments in PMP.

In addition, as shown in Figure 2b, the homopolymers PMMA and P(PEGDA) were completely dissolved in the THF and KOH solution, respectively. However, the PMP swelled and remained in an intact shape in either the THF or KOH solution, implying that the cross-linked network structure for PMP was formed. This cross-linked copolymer network could provide the polymer with a stationary phase and a reversible phase, and the polymer could change shape in response to thermal stimuli [17].

### 3.2. Shape Memory Behavior of PMP

**Figure 3:**
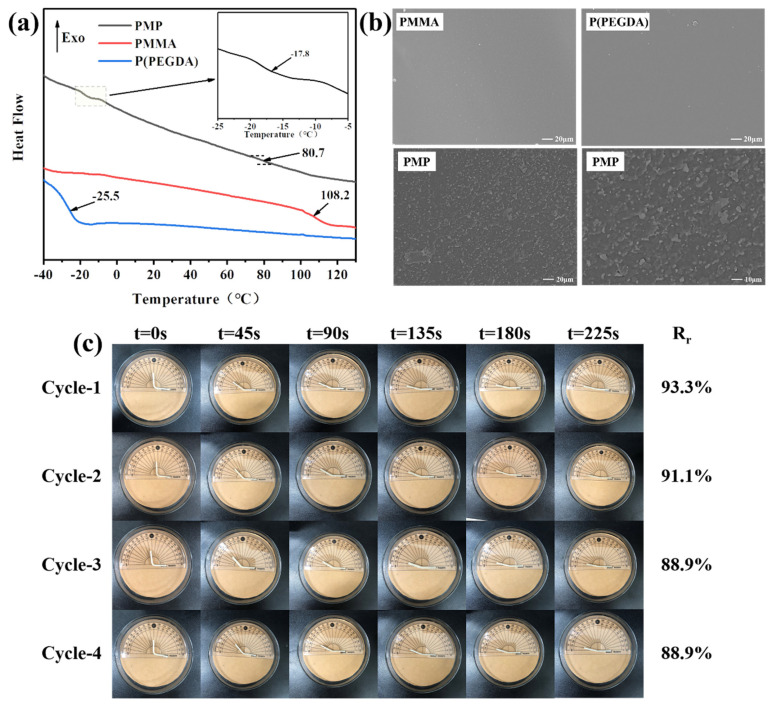
(**a**) DSC thermograms of PMMA, P(PEGDA) and PMP. (**b**) SEM micrographs of the fracture surface of PMMA, P(PEGDA) and PMP at different magnifications. (**c**) Cyclic shape memory behavior of PMP at 60 °C.

To better understand the effect of SMP on the dimensional stability of wood, the shape memory behavior of PMP was investigated. To select a suitable transition temperature (*T*_trans_) for studying the shape memory behavior of PMP, the glass transition temperature (*T*_g_) of the sample was detected by DSC measurement (Figure 3a). Both PMMA and P(PEGDA) showed a single *T*_g_ at 108.2 °C and −25.5 °C, respectively, while PMP had a double *T*_g_ at 80.7 °C and −17.8 °C [42,43]. This was consistent with the phase separated structure presented by the SEM images, as shown in Figure 3b. A sea-island structure with white spherical convex PEGDA-rich parts dispersed in a gray PMMA matrix was observed for the PMP sample. In contrast, no structure was observed in the SEM images of homopolymer PMMA and P(PEGDA). The lower *T*_g_ in PMP could be ascribed to the PEGDA-enriched segments, for example, the low molecular weight of P(PEGDA) and cross-linked PEGDA segments, comparatively; the higher *T*_g_ was probably sourced from the copolymer P(MMA-PEGDA), which was not as high as the *T*_g_ of PMMA [34,44].

The phase separation behavior of PMP leads to a wide range of softening temperatures. The most extreme temperature applied to wood generally reaches as high as 60 °C, which causes the large and drastic shrinking of wood, leading to cracks and splits. Fortunately, PMP contained a double *T*_g_ at 80.7 °C and −17.8 °C, and it was found that PMP could be deformed well and recovered to a great extent around 60 °C; consequently, the transition temperature (*T*_trans_) for conducting the shape memory behavior was fixed at 60 °C. When the temperature was set at 60 °C, which was much higher than the lower *T_g_* of PMP but below the higher *T*_g_ value of 80.7 °C, the switching segments were activated by softening and the original straight sample could be bent into an L-shape under the influence of external forces. The L-shape of the sample was temporarily fixed after cooling and was recovered to the original straight shape upon reheating. It was shown in the literature that the secret behind the SMP lies in its molecular network structure consisting of switches and netpoints [45]. Herein, for the sample of PMP, the switches responsible for shape fixation and partial shape recovery were the soft segments of PEGDA, while the netpoints that determined the permanent shape were likely to be the MMA/PEGDA copolymer. The shape fixity of PMP was determined to be as low as 66%, which could be ascribed to the low *T*_g_ of the PEGDA-rich segments phase.

Multiple cycles of the shape memory behavior of PMP were conducted at 60 °C and the results are shown in Figure 3c. As can be seen from the results, in four cycles, the shape of PMP recovered rapidly in the initial 45 s, with a recovery rate of 53.5% to 57.8%, and then slowed down during the rest of time. When the PMP specimens were softened and bent into an L-shape, entropic energy was stored via a chain conformation change. Upon reheating, the entropic energy was released, and as a result the shape recovered [46,47]. The continuous release of potential energy in the early stage of recovery resulted in a decline in the driving force for shape recovery; consequently, the shape recovery rate diminished gradually with time [48]. As the number of fixation-recover cycles increased, the recovery rate reduced slightly from 93.3% to 88.9%, which could be explained by the unrecoverable residual strain sourced from the relaxation of the chain conformation and probably the tiny slips of chain segments [47,49]. However, the overall response rate of PMP in multiple cycles remained at a high level, which is conducive to broadening the application scenarios of PMP.

### 3.3. In Situ Construction of PMP in Wood

**Figure 4:**
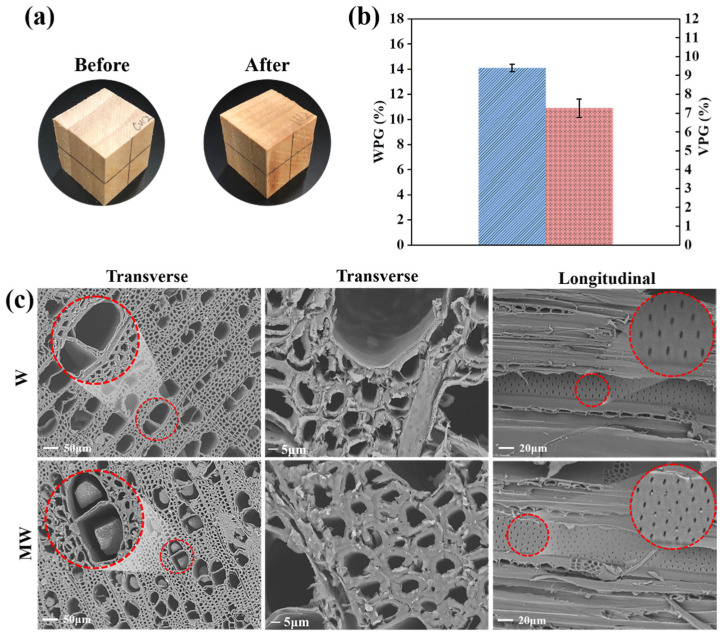
(**a**) Wood specimen before and after modification. (**b**) *WPG* and *VPG* of modified wood (MW). (**c**) SEM micrographs of untreated (W) and modified wood (MW).

Poplar is a widely distributed fast-growing wood that is high in permeability but poor in dimensional stability. To enhance the performance of poplar against water and moisture, PMP was in situ constructed within it. The color of the wood changed slightly after treatment, as shown in Figure 4a. The percentages of weight gain (*WPG*) and volume gain (*VPG*) are displayed in Figure 4b. The *WPG* and *VPG* reached as high as 14.0% and 7.2%, respectively, which could be contributed to the small molecular weight of the modifier and the efficiency of the reaction in the designed conditions.

The morphology of W and MW in Figure 4c showed that the polymers formed and filled in the cell cavities. In comparison with the untreated wood, the modified wood displayed swelled cell walls, densely packed with few spaces between the cells. For the treated samples, most of the pits in the vessel were also stuffed with white polymer, which suggested that a large amount of modifier solution penetrated the wood via the vessels and further went through the pits to the adjacent cells [37,50]. As the modifier solution entered the cell wall and polymerized, the cells would bulk and swell, which was confirmed by the *VPG* of the treated wood. The bulked cell wall of the wood fibers is shown in Figure 4c. The partially blocked cell cavities and pits could restrain the exchange of water and moisture to some extent, and accordingly enhance the dimensional stability of the wood.

### 3.4. Dimensional Stability of the Wood

**Figure 5:**
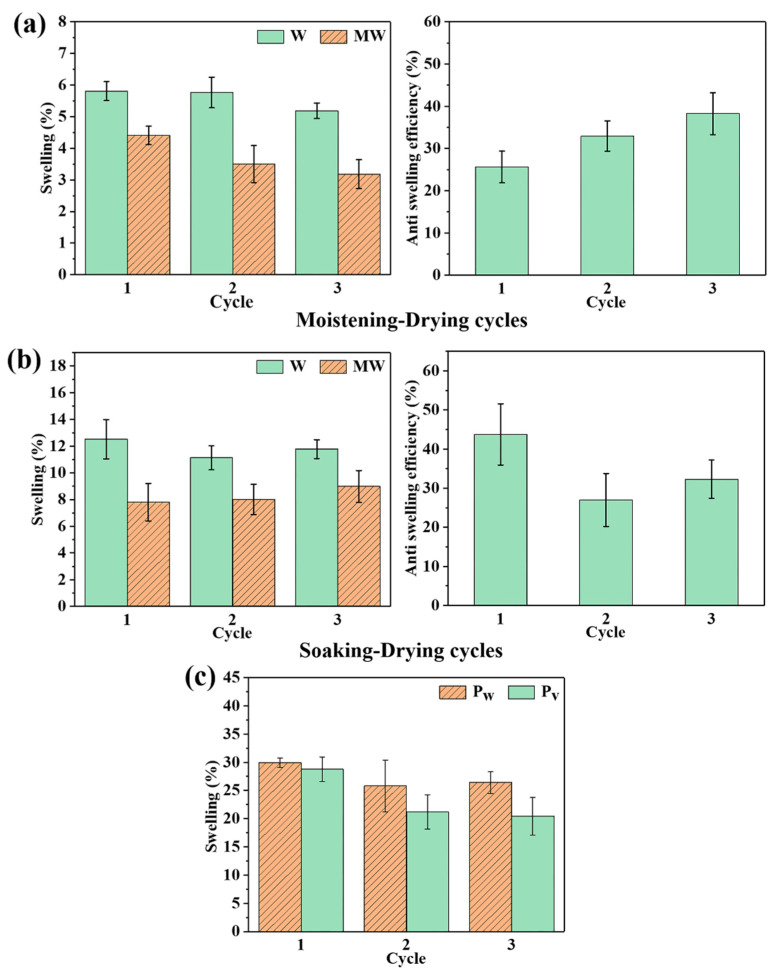
(**a**) The moisture absorption expansion rate and moisture absorption anti-swelling efficiency of the wood block, (**b**) the water absorption expansion rate and water absorption anti-swelling efficiency of wood block, and (**c**) the *P_w_* and *P_v_* of PMP during the three-cycle immersion test.

The wood used outdoors is generally subjected to a changing climate; accordingly, an alternating dry and wet condition was designed to perform the wood dimensional stability test. The test was conducted under cyclic moistening-drying (M-D) and water soaking-drying (S-D) procedures. The swelling rate of SMP-treated wood was 4.41%, comparatively large, in the first M-D procedure. In the next two M-D cycles, the modified wood presented a decline in swelling rate and the anti-swelling efficiency (*ASE*) increased to 32.94% and 38.23% (Figure 5a). This phenomenon could be attributed to the reduced dimensional variation of the polymer-bulked cell wall, and was confirmed by the test on wood conditioning shown in Table 1.

When the oven-dried untreated (W) and modified wood (MW, MC < 1%) were placed in a conditioning vessel and the MC was increased to a constant value, a state of fiber saturation point (FSP) with MC 28.63% and 32.75% was reached, respectively. It is interesting to note that the increased volume of MW was 4.53% lower than W, although the former was 4.12% higher in MC than the latter, which suggested that the SMP-modified wood could maintain its volume upon absorbing a certain amount of moisture.

In the soaking procedure of the S-D cycles, the swelling rate of the modified wood was 7.80%, and the values of *ASE* were in the range of 26.98% to 43.77% (Figure 5b). The partially blocked pits arising from the modifier likely contributed to the reduced swelling [10]. It is worth noting that the modified wood exhibited almost constant swelling rates during the three S-D procedures, which could be attributed to the performance of the polymer in S-D procedures. The polymer had a low ratio of volume swell percentage (*P_v_*) to weight gain percentage (*P_w_*) in the S-D procedures (Figure 5c), which meant that the polymer could absorb more water with a relatively small volumetric change. The values of *P_v_*/*P_w_* dropped from 0.96 to 0.77 during the three S-D cycles, which was probably caused by the leaching of soluble and hydrophilic segments. The leaching of these segments can occur in SMP-modified wood, and as a result, the *ASE* of the SMP-modified wood increased slightly during the last two cycles [51].

**Figure 6:**
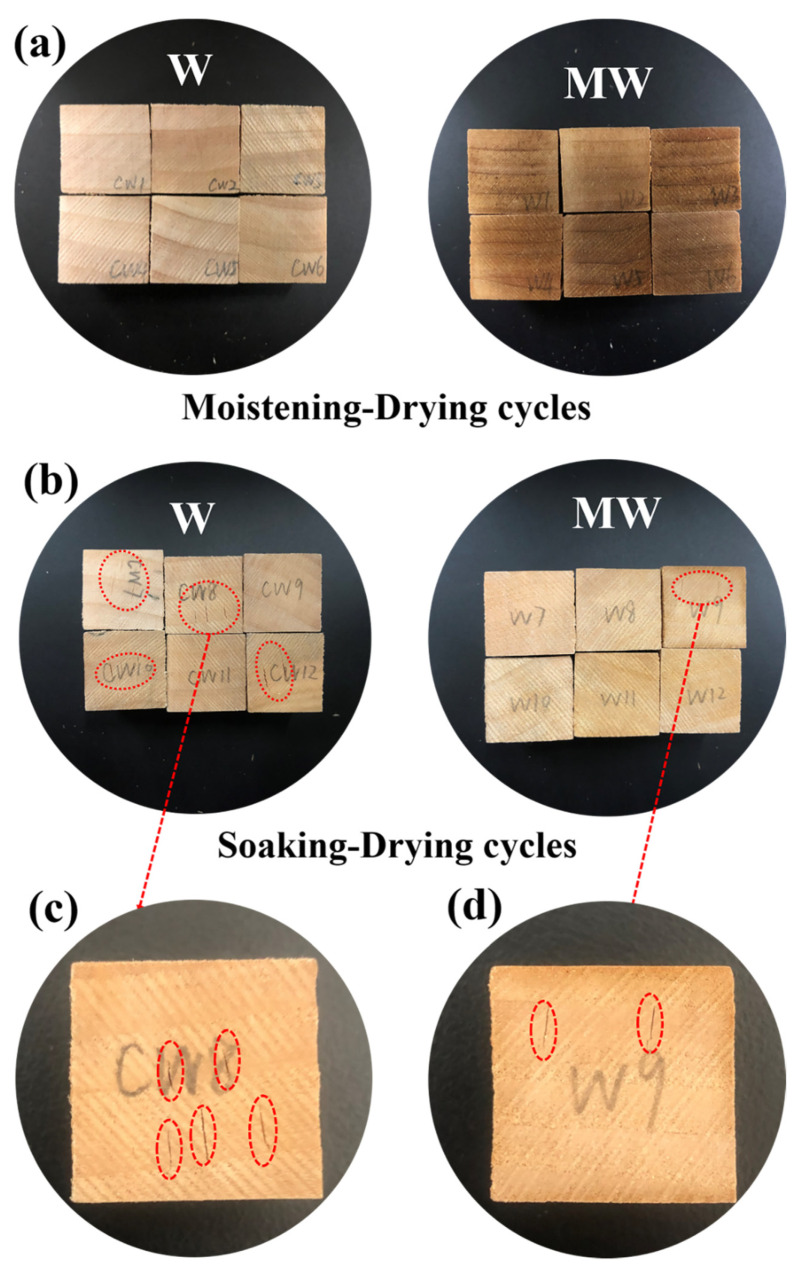
Digital photos of W and MW after moistening-drying cycles (**a**) and after soaking-drying cycles (**b**); (**c**,**d**) show higher magnifications for the cracked samples. The cracks are indicated by red circles.

Figure 6 shows the digital images of the samples of untreated (W) and modified wood (MW) after three cycles of moistening-drying and soaking-drying. During the cyclic M-D procedures, both MW and W behaved well, free from surface cracks (Figure 6a). However, they behaved completely different after three S-D cycles. As clearly presented in Figure 6b, most of the W specimens (four out of six) were cracked and a number of cracks were found on each wood surface, while very few cracks appeared on the MW blocks. Only one MW sample was cracked after S-D treatment. As many as five cracks appeared on one of the W samples, shown by the magnified image in Figure 6c, while the only cracked MW sample displayed two cracks (Figure 6d). This showed that poplar wood filled with SMP could effectively reduce the risk of cracks from internal stresses caused by shrinkage.

### 3.5. Mechanism of Action of SMP on Wood Dimensional Stability

**Figure 7:**
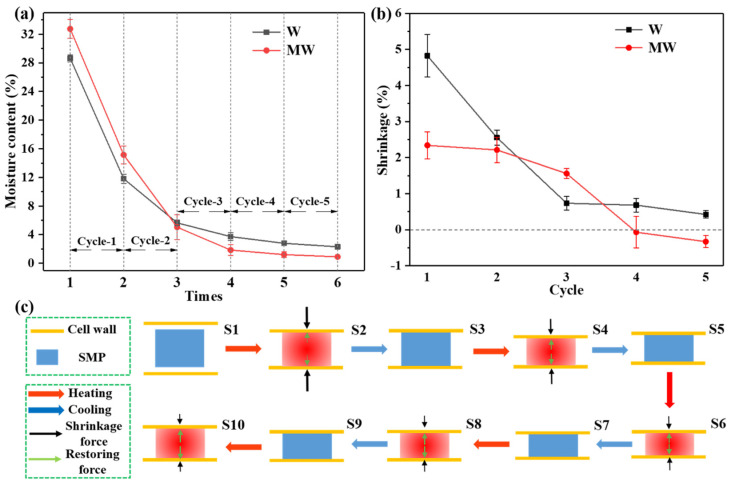
(**a**) The change curve of wood moisture content with heating-cooling cycle. (**b**) The shrinkage of W and MW. (**c**) Schematic illustration of SMP in the wood cell wall.

To reveal the mechanism of action of shape memory polymer (SMP) in stabilizing the wood dimensions, a continuous drying test was conducted. Firstly, both the W and MW were conditioned to the FSP state, and the MC for W and MW were determined as 28.63% and 32.75%, respectively (Figure 7a). Afterward, they were subjected to five cycles of 60 °C and 25 °C treatments; the recorded MC and volume variation are shown in Figure 7a,b.

As a whole, the loss of water under high temperature caused a significantly dimensional shrinkage of the untreated wood, accompanied by cracks, particularly during the first three cycles when the MC decreased from around 30% to 4%. After that, the change in dimension appeared to level off because the water in wood became difficult to remove. In comparison with the untreated wood, the modified wood displayed a stable and small variation in dimension when the MC dropped from 28.63% to 3.72%. The decrease in the dimensional change of MW could be attributed to the thermal-responsive SMP filled in wood cell wall, which retarded the shrinkage of the cell wall by thermal expansion at a high temperature. Moreover, the recovery of SMP counteracted the stress from the shrinking of the cell wall; as a result, the modified wood exhibited few cracks, as shown in Figure 6b [52,53]. It is worth noting that during the fourth and fifth cycles, the dimensional variation of the MW sample became negative, which suggested that the volume of MW expanded when heated. This phenomenon could also be explained by the characteristics of the SMP filled in the cell wall. As the MC of MW approached zero after three cycles, the shrinkage of the cell wall from reduced MC could be ignored, while SMP maintained its characteristics of shape fixation and recovery, which expanded the cell wall upon heating and, consequently, the wood showed an increased dimension. Nevertheless, the MC of wood almost never dropped to zero in practice.

The deformation and relaxation of SMP in the wood cell wall are schematically illustrated in Figure 7c, where S1–S10 represent step 1–10. After modification, the SMP is constructed in wood, staying in the initial state (Figure 7(c-S1)). When the temperature is increased to 60 °C, the water is lost, the wood will shrink and the polymer will be compressed to state 2 (Figure 7(c-S2)). As the temperature is dropped to 25 °C, a temporary stressed state 3 (Figure 7(c-S3)) will be reached. Upon heating, wood will shrink again, while the confined polymer tends to recover to its initial state; the reversed stress produced by the polymer will resist the stress from the wood shrinkage (Figure 7(c-S4)). As the two stresses contradict, the stress in wood will be partially released, which helps in restraining both the volumetric variation and cracking developed by stress accumulation. When it returns to a normal temperature, the polymer will go to a second temporary state with residual stress (Figure 7(c-S5)). Subsequently, when the modified wood undergoes repeated 60 °C and 25 °C procedures, accompanied by the reduced MC and shrinkage of wood, the stored stress in SMP will be accumulated, which will ultimately be released and expand the cell wall to some extent (Figure 7(c-S10)).

## 4. Conclusions

A cross-linked copolymer network with shape memory behavior was fabricated by reacting methyl methacrylate (MMA) and polyethylene glycol diacrylate (PEGDA) in a water/ethanol solution. Their cross-linking characteristics were evidenced by the FT-IR and dissolution analysis. The phase separation of PMP was observed with the help of SEM. A relatively wide glass transition temperature range, i.e., the double *T*_g_ of PMP at −17.8 °C and 80.7 °C, was found by the DSC measurement, based on which the shape memory performance pof PMP was studied. An alternating 60 °C and 25 °C cycle was designed to simulate high temperature sunny days and cooling days. Under the variation of temperature, a desirable shape memory performance was detected for the PMP, which retarded the rapid shrink of in situ constructed PMP-modified wood under high temperature. PMP was found to be distributed mainly in the cell wall, cell cavity and the pits. The presence of PMP in these pores not only retarded the transportation and exchange of water but also counteracted the internal stress and shrinkage of the wood accompanied by water evaporation. The variation and function of internal force will be researched in forthcoming studies.

## Figures and Tables

**Table 1:** Volumetric change of wood samples under different moisture content.

Samples	MC_0_ (%)	V_0_ (cm^3^)	MC_1_ (%)	V_1_ (cm^3^)	Volumetric Change (%)
W	0.50	8.21 ± 0.26	28.63	9.35 ± 0.27	13.90
MW	0.61	8.45 ± 0.23	32.75	9.24 ± 0.24	9.37

Note: W and MW represent the untreated and modified wood, respectively; MC_0_ and MC_1_ represent the wood that was dried or conditioned to an absolutely dry state and the fiber saturation point, respectively; V_0_ and V_1_ correspond to the volume of the test block at the two states of moisture content. Volumetric change represents the rate of volume change from V_0_ to V_1_.
